# Recent Advances in Basic Research for CSF1R-Microglial Encephalopathy

**DOI:** 10.3389/fnagi.2021.792840

**Published:** 2021-12-09

**Authors:** Yan-Li Wang, Fang-Ze Wang, Runzhi Li, Jiwei Jiang, Xiangrong Liu, Jun Xu

**Affiliations:** ^1^Department of Neurology, Beijing Tiantan Hospital, Capital Medical University, Beijing, China; ^2^Department of Cardiology, Weifang People’s Hospital, Weifang, China; ^3^China National Clinical Research Center for Neurological Diseases, Beijing Tiantan Hospital, Capital Medical University, Beijing, China

**Keywords:** CSF1R-microglial encephalopathy, microglia, dementia, mutation, pathophysiological mechanism, microglial replacement

## Abstract

Colony-stimulating factor-1 receptor-microglial encephalopathy is a rare rapidly progressive dementia resulting from colony-stimulating factor-1 receptor (CSF1R) mutations, also named pigmentary orthochromatic leukodystrophy (POLD), hereditary diffuse leukoencephalopathy with spheroids (HDLS), adult-onset leukoencephalopathy with axonal spheroids, and pigmented glia (ALSP) and CSF1R-related leukoencephalopathy. *CSF1R* is primarily expressed in microglia and mutations normally directly lead to changes in microglial number and function. Many animal models have been constructed to explore pathogenic mechanisms and potential therapeutic strategies, including zebrafish, mice, and rat models which are with *CSF1R* monogenic mutation, biallelic or tri-allelic deletion, or *CSF1R*-null. Although there is no cure for patients with CSF1R-microglial encephalopathy, microglial replacement therapy has become a topical research area. This review summarizes *CSF1R*-related pathogenetic mutation sites and mechanisms, especially the feasibility of the microglia-original immunotherapy.

## Introduction

Colony-stimulating factor-1 receptor -microglial encephalopathy, microglia-original dementia, is a rare autosomal dominant disease caused by mutations in the colony-stimulating factor-1 receptor (CSF1R) gene resulting in microglial dysfunction. Clinically, it is manifested by progressive cognitive decline, motor impairment accompanied by mental behavioral abnormalities. CSF1R-microglial encephalopathy typically presents as rapidly progressive dementia. The peak age of onset is 8–72 years (mean 42 years) and the prognosis is poor with a median survival of 2–30 years (mean 6 years) (Lynch et al., 2019). This review discusses the pathophysiology, corresponding animal models, and management options of CSF1R-microglial encephalopathy to promote clinical awareness.

## Historical Background and Nomenclature

**FIGURE 1:**
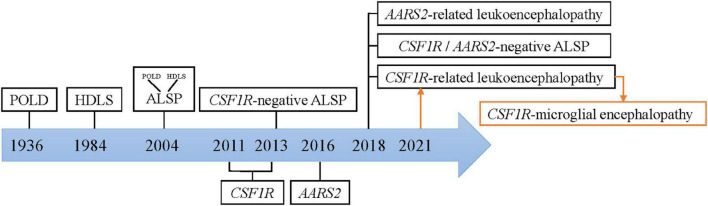
Nomenclature of CSF1R-microglial encephalopathy.

Colony-stimulating factor-1 receptor (CSF1R)-microglial encephalopathy was also known as pigmentary orthochromatic leukodystrophy (POLD), hereditary diffuse leukoencephalopathy with spheroids (HDLS), adult-onset leukoencephalopathy with axonal spheroids, and pigmented glia (ALSP), and CSF1R-related leukoencephalopathy. The naming process was shown in Figure 1.

The study Marotti et al. (2004) found that there are many similarities between the original brain sections of the first POLD diagnosed in 1936 and the brain autopsy sections of HDLS families confirmed in 1984, including white matter degeneration, corpus callosum atrophy, pigmented glia, neuroaxonal spheroids and demyelination, and so forth. Besides, the clinical manifestations of POLD are quite similar to HDLS. Thus, it is inferred that POLD and HDLS to be the same disease (i.e., ALSP). The diagnostic criteria for these rare diseases are mostly based on sporadic cases and small case series. Therefore, rare diseases are easily classified as the same disease, when with similar clinical and pathological features. And known nomenclature might simply be an expression of pathological changes, ignoring microglia-original abnormalities.

In recent years, genome-wide association (GWAS) and whole exome/genome sequencing (WES/WGS), have been widely used for mutational analysis in rare diseases. With a common causal gene CSF1R being identified, HDLS and POLD were reconfirmed to be the same disease. And this evidence provides genetic evidence for ALSP diagnostics (Rademakers et al., 2011; Nicholson et al., 2013). But no mutations in the CSF1R gene were found in the first confirmed family of HDLS in 1984 (Sundal et al., 2013). Besides, many patients have typical ALSP clinical manifestations, including progressive cognitive decline, motor impairment, and mental behavioral abnormalities, but no CSF1R mutation (Lynch et al., 2016). Therefore, ALSP is further divided into CSF1R- related leukoencephalopathy, AARS2- related leukoencephalopathy, and CSF1R/AARS2-negative ALSP (Konno et al., 2018). Currently, whether leukodystrophy is the same concept as leukoencephalopathy remains controversial. Microglial dysfunction caused by mutations in the CSF1R gene has been put forward as the fundamental pathogenetic mechanism of this rare disease (Prinz and Priller, 2014). Hence, this review summarized the current basic research with the term “CSF1R-microglial encephalopathy.”

## CSF1R Mutation Sites

**FIGURE 2:**
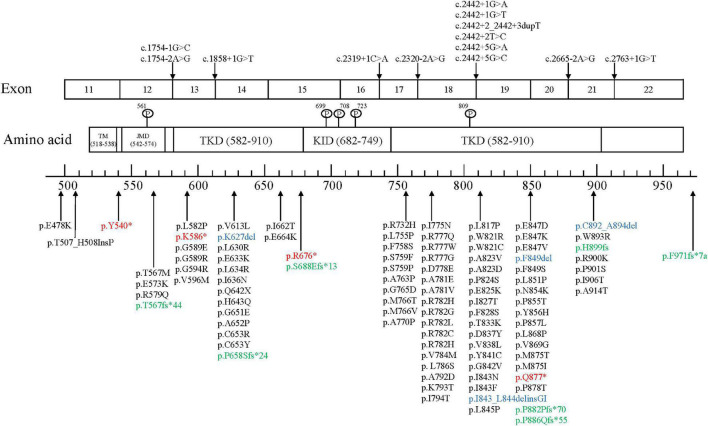
*CSF1R* mutation sites.

The colony-stimulating factor-1 receptor (*CSF1R*) gene is located on chromosome 5q32 and includes 22 exons that encode cellular membrane proteins. CSF1R is a receptor tyrosine kinase made up of 5 functional domains: 5 immunoglobulin-like motifs in the extracellular domain, a transmembrane domain (TM), a juxtamembrane domain (JMD), a kinase insertion domain (KID), and dichotomous tyrosine kinase domains (TKDs). Most gene mutations are located at the tyrosine kinase domain of CSF1R encoded by exons 12–21, and no disease-associated mutations located in exon 16 have been discovered yet. As of October 2021, a total of 114 mutation sites has been reported globally, including 93 missense mutations, 4 nonsense mutations (red), 4 insertions or deletions (blue), 7 frameshift mutations (green), and 13 splice-site mutations (Figure 2; Konno et al., 2018; Lai et al., 2020; Zhuang et al., 2020; Ayrignac et al., 2021; Chu et al., 2021; Du et al., 2021; Kındış et al., 2021; Sohn et al., 2021; Tipton et al., 2021; Tsai et al., 2021).

## Pathophysiological Mechanisms

In the central nervous system, CSF1R is predominantly expressed on microglia. *CSF1R* knockout leads to a decrease in the number of microglia and exists a high spatial heterogeneity (Prinz and Priller, 2014). Cerebellar CSF1R expression seems to be low when compared with cortex, hippocampus, and striatum in Csf1r-EGFP transgenic mice (Hawley et al., 2018). *In vitro* studies have found that *CSF1R* mutations would lead to autophosphorylation of tyrosine residues lost through two mechanisms, specifically one possibility is a dominant-negative mechanism and the other with loss of function. Mutations located in the juxtamembrane domain and the kinase insert region, which assembles into homodimers and heterodimers, resulting in the inhibition of kinase activity, thereby suppressing phosphorylation of its downstream targets (Rademakers et al., 2011; Leng et al., 2019). Mutations located in the tyrosine kinase domain, which result in the inactivation of a tyrosine kinase, fail to continue signal transduction (Pridans et al., 2013).

The haploinsufficiency of the *CSF1R* gene could result in the lack of microglia in the *CSF1R*-null zebrafish model, including an overall decrease of microglia and a region-specific reduction (Oosterhof et al., 2019). Based on RNA sequencing results for zebrafish of wide-type, biallelic deletion and tri-allelic deletion of *CSF1R*, lack of CSF1R might also up-regulate genes related to immune response processes, including chemokine genes and chemokine receptor genes and down-regulated genes related to nervous system development and neuronal differentiation (Oosterhof et al., 2018). Besides, changes in CSF1R could affect microglial distribution, so the abnormal distribution of microglia is clearly seen in the *CSF1R* tri-allelic deletion of the zebrafish model (Oosterhof et al., 2018). The results of necropsy indicated that residual microglia had a strong proliferative ability though the numbers of microglia in specific brain regions were significantly reduced (Tada et al., 2016).

The *CSF1R* gene expression is increased in response to brain injury marginally expressed in the hippocampus, neurons and neural stem cells, this suggests a neuroprotective role for CSF1R (Luo et al., 2013). Systemic Csf1r-knockout mice can exhibit osteopetrosis, reduction in marrow hematopoiesis, and defection in reproductive function, impairment in olfactory capacity, and reduction in mononuclear phagocyte, including microglia (Dai et al., 2002; Li et al., 2006; Chitu et al., 2016). And the most prominent difference was the length of survival time. *CSF1R*-null mice only had a median survival of 6 weeks, and a significant reduction of microglia is the most obvious feature additionally with enlarged ventricle and skeletal dysplasia (Guo et al., 2019). Because the early lethality of this model is high, the *CSF1R*-null mice model is not suitable to investigate neurodegenerative disease. Although the conditional Csf1r-knockout mice can selectively suppress CSF1R expression and make microglia absent, the mouse phenotype is not clinically consistent with CSF1R-microglial encephalopathy (Rojo et al., 2019). Homozygous infants with *CSF1R* deletion suffer death within 1 year of birth, accompanied by microglial deficiency, macrocephaly, and osteopetrosis (Oosterhof et al., 2019). Microglia are fully absent in the *CSF1R*-null rat model showed growth retardation, skeletal abnormality, and infertility, but most brain structural without abnormality and the rat can survive to adulthood (Guo et al., 2019). Therefore, the *CSF1R*-null rat model poses a possibility for long-term follow-up effects of different interventions.

It is also found in the *CSF1R*-null zebrafish model that the expression of neuronal transcription factor CUX1 is significantly decreased (Oosterhof et al., 2019). Additionally, CUX1 is a transcription factor associated with axonal projections indicating that reducing the number of CUX1+ neurons may lead to the hypoplastic corpus callosum (Oosterhof et al., 2019). Mice with *CSF1R* ± monogenic mutation are viable, fertile, and without skeletal abnormalities, but then may present cognitive decline, depression, and anxiety (Guo et al., 2019). It is found that *CSF1R* interacts with *TRME2* through the transmembrane region and thereby regulates each other by Cheng et al. (2021). *CSF1R* knockdown markedly increases the *TREM2* mRNA levels and *TRME2* suppression results in elevated mRNA and protein levels of CSF1R. Thus, the phenotype of Trem2-deficient microglia can be rescued by activating CSF1R signaling (Cheng et al., 2021). It has been reported, CSF2 expression is increased in both the Csf1r± mice model and patients with *CSF1R*-microglial encephalopathy (Chitu et al., 2020). Targeted disruption of the murine Csf2 allele could suppress microgliosis, inhibit oxidative stress and improve microglial dysfunction, and ameliorate spatial memory, depression-like behavior, olfactory dysfunction, and motor coordination (Chitu et al., 2020).

## Microglial Replacement Therapy

Experimental mouse models of reduced *CSF1R* expression levels indicate that the absence of microglia could contribute to repopulated niche by the newly microglia. However, the source of the repopulated microglia is yet to be fully confirmed. Using different modified mouse lines, Huang et al. (2018) demonstrate that the repopulated microglia is from the proliferation of surviving microglia (<1%). Furthermore, based on findings of *Cx3cr1^CreER/+^R26^DTA/+^* mice, microglia-like cells could be produced by infiltration of peripheral monocytes into the brain (Lund et al., 2018). Both types are not mutually exclusive and can happen at the same time.

## Based on the Proliferation of Resident Microglia

Specific inhibitors of CSF1R target and purge 99% microglia in the CNS and cross the blood-brain barrier, but neither mouse abnormal behavior nor cognitive decline was observed. Microglial numbers return to normal levels 1 week after inhibitor withdrawal (Elmore et al., 2014). After a 28-day repopulation, microglial morphology has been reproduced rejuvenation. And dendrite complexity has been recovered in hippocampal neurons of 24-month mice (Elmore et al., 2018). Microglial repopulation contributes to reversing the expression of all these genes related to actin cytoskeleton remodeling and synaptogenesis, inducing neurogenesis and ameliorating age-related memory impairment (Elmore et al., 2018; Willis et al., 2020). Furthermore, some trials of PLX5622 have found that small molecule inhibitors can affect not only brain microglia, but also bone marrow-derived macrophages, tissue macrophages, and circulating monocytes (Lei et al., 2020). Therefore, the application of CSF1R inhibitor to patients with CSF1R-microglial encephalopathy should be cautiously considered. A study, in which adult CSF1R± mice were treated with persistent low-grade PLX5622 at different stages of the disease, has found that modulating microglial phenotypes could reverse presynaptic and ECM alterations induced by *CSF1R* haploinsufficiency and improved cognition without altering neuronal populations. This finding suggests that microglia are viable targets for therapeutic intervention early in the disease (Arreola et al., 2021).

## Based on Transplantation of Microglia-Like Cells

Microglia-like cells differentiated by allogeneic bone marrow transplantation (BMT) have the option to replace 92.66% of resident microglia in the brain. Normally, microglia replacement by BMT (mrBMT) remains at resting-state. While in response to inflammatory stimuli, microglia-like cells are activated performing the immune function (Xu et al., 2020). Peripheral blood-derived microglia-like cells (mrPB) can replace 80.74% of resident microglia, and acquire mature phenotype to monitor intracellular environmental stabilization after 30 days (Xu et al., 2020). Single-cell RNA sequencing reveals that microglia-like cells have macrophage-like properties, which is different from the yolk sac-derived CNS resident microglia epigenetically (Lund et al., 2018; Xu et al., 2020). The novel replacement therapies are suitable for neonatal and adult mice. Clinically, bone marrow transplantation and peripheral blood graft hold promise for the treatment of CSF1R-microglial encephalopathy.

## Based on Hematopoietic Stem Cell Transplantation

Microglia replacement by exogenous microglia transplantation (mrMT) shares very similar morphology and RNA expression profiling of resident microglial (Xu et al., 2020). It is difficult to acquire sufficient endogenous microglia for therapeutic transplantation, so with the help of hematopoietic stem cell transplantation (HSCT), protein expression is normalized for CSF1R in replacement wide-type microglia and the inhibited CSF1R pathways are activated. Patients achieve stable disease with relatively stable expanded disability status score and decreased volume of white matter hyperintensities 6–30 months after undergoing transplantation (Mochel et al., 2019). If HSCT could be performed early in the disease, survival could extend beyond 15 years and patients may have normal communication abilities (Eichler et al., 2016). Donor chimerism reaches 100%, 9 months after transplantation, besides, cognitive and motor impairment are markedly improved and hyperintensities of the foci completely regress after 28 months (Gelfand et al., 2020). And stabilizing effects of HSCT have been found in the largest longitudinal study of 7 patients with CSF1R-microglial encephalopathy receiving HSCT, containing motor capacity, cognition, radiographic severity, and white matter lesion burden (Tipton et al., 2021).

## Conclusion and Outlook

At present, works of research on the mechanism of CSF1R-microglial encephalopathy are still few and lack animal models with missense mutations. The emerging microglial replacement therapy is a reliable modality to relieve clinical symptoms and extend survival. But further experimental studies and clinical trials with long-term follow-up are still needed to assess potential risks.

## Author Contributions

JX and XL conceived and designed the study. Y-LW and F-ZW contributed to the generation of the manuscript. RL and JJ helped revise the manuscript. All authors contributed to the editing of the manuscript.

## Conflict of Interest

The authors declare that the research was conducted in the absence of any commercial or financial relationships that could be construed as a potential conflict of interest.

## Publisher’s Note

All claims expressed in this article are solely those of the authors and do not necessarily represent those of their affiliated organizations, or those of the publisher, the editors and the reviewers. Any product that may be evaluated in this article, or claim that may be made by its manufacturer, is not guaranteed or endorsed by the publisher.
